# Factors Affecting the Adoption of Artificial Intelligence-Enabled Virtual Assistants for Leukemia Self-Management

**DOI:** 10.7759/cureus.49724

**Published:** 2023-11-30

**Authors:** Turki Alanzi, Reham Almahdi, Danya Alghanim, Lamyaa Almusmili, Amani Saleh, Sarah Alanazi, Kienaz Alshobaki, Renad Attar, Abdulaziz Al Qunais, Haneen Alzahrani, Rawan Alshehri, Amenah Sulail, Ali Alblwi, Nawaf Alanzi, Nouf Alanzi

**Affiliations:** 1 Department of Health Information Management and Technology, College of Public Health, Imam Abdulrahman Bin Faisal University, Dammam, SAU; 2 College of Medicine, Al Baha University, Al Baha, SAU; 3 College of Medicine and Surgery, Royal College of Surgeons in Ireland, Dublin, IRL; 4 College of Pharmacy, Jazan University, Jazan, SAU; 5 Faculty of Pharmacy, Ibnsina National College of Medical Studies, Jeddah, SAU; 6 Department of Pharmacy, Almoosa Specialist Hospital, Al Mubarraz, SAU; 7 College of Medicine, King Abdulaziz University, Jeddah, SAU; 8 College of Medicine, Imam Abdulrahman Bin Faisal University, Dammam, SAU; 9 Department of Hematology, Armed Forces Hospital at King Abdulaziz Airbase Dhahran, Dhahran, SAU; 10 College of Medicine, Taif University, Taif, SAU; 11 College of Public Health, Imam Abdulrahman Bin Faisal University, Dammam, SAU; 12 Department of Blood Bank, Regional Laboratory and Blood Banks Arar, Arar, SAU; 13 Department of Clinical Laboratory Sciences, College of Applied Medical Sciences, Jouf University, Jouf, SAU

**Keywords:** benefits, challenges, technology acceptance, cancer, virtual assistants, leukemia, artificial intelligence

## Abstract

Aim and purpose: The purpose of this study is to analyze the various influencing factors affecting the adoption of artificial intelligence (AI)-enabled virtual assistants (VAs) for self-management of leukemia.

Methods: A cross-sectional survey design is adopted in this study. The questionnaire included eight factors (performance expectancy, effort expectancy, social influence, facilitating conditions, behavioral intention, trust, perceived privacy risk, and personal innovativeness) affecting the acceptance of AI-enabled virtual assistants. A total of 397 leukemia patients participated in the online survey.

Results: Performance expectancy (μ = 3.14), effort expectancy (μ = 3.05), and personal innovativeness (μ = 3.14) were identified to be the major influencing factors of AI adoption. Statistically significant differences (p < .05) were observed between the gender-based and age groups of the participants in relation to the various factors. In addition, perceived privacy risks were negatively correlated with all other factors.

Conclusion: Although there are negative factors such as privacy risks and ethical issues in AI adoption, perceived effectiveness and ease of use among individuals are leading to greater adoption of AI-enabled VAs.

## Introduction

Leukemia, a complex and life-threatening blood cancer, poses significant challenges to patients and healthcare systems worldwide [1,2]. The management of leukemia necessitates continuous monitoring, timely interventions, and informed decision-making to improve patient outcomes [3,4]. In recent years, artificial intelligence (AI) has emerged as a powerful tool in healthcare, offering promising solutions for better disease management and patient care. AI-enabled virtual assistants (VAs) have garnered attention as a potential means of providing self-help and enhancing leukemia management [5].

The adoption of AI in healthcare is driven by several key factors. Technological advancements, including machine learning algorithms and natural language processing, have empowered AI systems to understand and respond to human queries with unprecedented accuracy. Moreover, the proliferation of smartphones and smart devices has created a fertile ground for integrating virtual assistants into patients' daily routines [6]. The coronavirus disease 2019 (COVID-19) pandemic has further accelerated the adoption of remote healthcare solutions such as mHealth and eHealth applications [7], making AI VAs an attractive option for leukemia patients who may need to minimize in-person clinic visits.

However, the successful adoption of AI VAs in leukemia management is not without its obstacles [8]. Concerns about data privacy and security, the need for user-friendly interfaces, and the role of healthcare professionals in overseeing AI-driven care all present challenges that must be addressed. Additionally, patient trust in AI-driven solutions is paramount, and understanding the factors that influence this trust is a critical aspect of our investigation. Empirical research on the adoption of AI-enabled VAs has found various influencing factors, whose impact varied across the studies. A recent study [9] focusing on the determinants to accept VAs among cancer patients has identified that performance expectancy (PE: the level that the individual believes that the use of the system can help him gain benefits in his activities), effort expectancy (EE: the level of ease of use associated with the use of a system), social influence (SI: an individual feels the importance that the others believe he or she should use the new system), and trust (a willingness to depend on the specific technology in a given situation in which negative consequences are possible) [10] significantly influenced behavioral intention (BI) to use VAs. However, facilitating conditions (FC: the degree to which an individual believes that an organization’s and technical infrastructure exists to support the use of the system) was identified to be not having a significant relationship with BI. Furthermore, lack of knowledge and awareness about AI technologies, their risks, and challenges were identified to be a few major influencing factors affecting the use of AI-assisted technologies among patients and physicians [11-13]. In a similar study [14], trust and personal innovativeness (PI: the individual's propensity and willingness to explore and examine new technologies and innovations) were identified as positively influencing intention to use VAs; while PE, EE, FC, SI, and perceived privacy risks (PPR) did not have any significant effect on the BI to use VAs. Similarly, in another study [15] focusing on the use of VAs for improving mental health, it was observed that PE and distress were found to be positive influencing factors of adopting VAs; whereas EE and AI hesitancy were identified to be negatively correlated with adoption of VAs. Furthermore, socio-technical factors like experience with technology, cultural factors, and socio-economic status were identified to be influencing the adoption of innovative technologies in healthcare [16]. These findings indicate that the impact of different factors on the BI to use VAs varied across the populations, indicating the need to further explore the area in different settings to gain a better understanding of the acceptance of VAs for self-health management. Accordingly, this study aims to investigate the influencing factors of AI-enabled VA adoption for self-help and management of leukemia.

In this comprehensive study, the multifaceted landscape of AI-enabled VA adoption for leukemia management is explored. Patient attitudes and perspectives, and the technical and ethical considerations that underpin the adoption process are investigated. By shedding light on these influencing factors, valuable insights into the future of AI-driven self-help and leukemia management are derived, ultimately striving for improved patient outcomes and enhanced healthcare delivery in the face of this challenging disease.

## Materials and methods

Recruitment and sampling

The participants in this study included leukemia patients recruited from public hospitals and social self-help communities. As participants are purposively recruited from the selected institutions, convenience and purposive sampling techniques were adopted [17]. The inclusion criteria included adult leukemia patients who have been using or are aware of AI-powered VAs for self-help and management of their condition.

Instruments

The survey questionnaire is divided into two sections. The first section focuses on collecting demographic information related to age, gender, education, and experience with AI-assisted technologies. The second section focuses on collecting the data on AI technology influencing factors. This study has adopted four factors including performance expectancy (four items), effort expectancy (three items), social influence (three items), and facilitating conditions (four items) from [18,19]. In addition, behavioral intention (three items) was adopted from [20]. In addition, three factors including perceived privacy risks (four items), trust (four items), and personal innovativeness (four items) were adopted from [14]. The questionnaire was designed using Google Forms, by creating a link to access the survey. A pilot study was conducted with 14 physicians, and the data was analyzed. Cronbach alpha was calculated for all items and was observed to be greater than 0.7, indicating good internal consistency [21].

Ethical considerations

All the participants were fully informed about the study through an information sheet attached to the invitation email. An informed consent was taken from all the participants using a check button, before starting the survey. The participation was voluntary and the participants were assured of their anonymity and their rights with respect to the data. Ethical approval was received from the ethics committee at Imam Abdulrahman Bin Faisal University (IRB-2023-03-417; Dt: 24/10/23).

Data collection

A participant information sheet is attached along with the invitation email (containing a survey link), explaining the rights of the participants, and forwarded to all the patients who agreed to participate in the survey. A total of 438 patients participated in the survey. However, 41 responses were incomplete. After cleaning the data, a total of 397 patient responses were considered for data analysis.

Data analysis

To attain the objectives of the research, the researcher utilized the Statistical Package for the Social Sciences (SPSS, version 24; IBM Corp., Armonk, NY, USA) for analyzing the data. Descriptive statistics will be used to characterize the participants’ demographic data. In addition, two-sample t-tests with unequal variances and single-factor ANOVA were used for analyzing the data. Furthermore, Pearson correlation coefficients were used to compare the relationship between various factors.

## Results

As shown in Table 1, a total of 397 leukemia patients participated in the study, with appropriate representation of both genders (58.7% males and 41.3% females). Among the participants, 75% were aged below 41 years and 25% were aged 41 or more years. The majority of the participants had a diploma degree (40.6%), followed by bachelor’s degree (24.4%).

**Table 1:** Participants demographics

Variable		N	Relative frequency
Age (in years)	18-30	157	39.5%
	31-40	141	35.5%
	41-50	86	21.7%
	51-60	13	3.3%
Gender	Male	233	58.7%
	Female	164	41.3%
Education	Diploma	161	40.6%
	Bachelor’s degree	97	24.4%
	Master’s degree	75	18.9%
	Ph.D.	15	3.8%
	Other	49	12.3%

**Figure 1:**
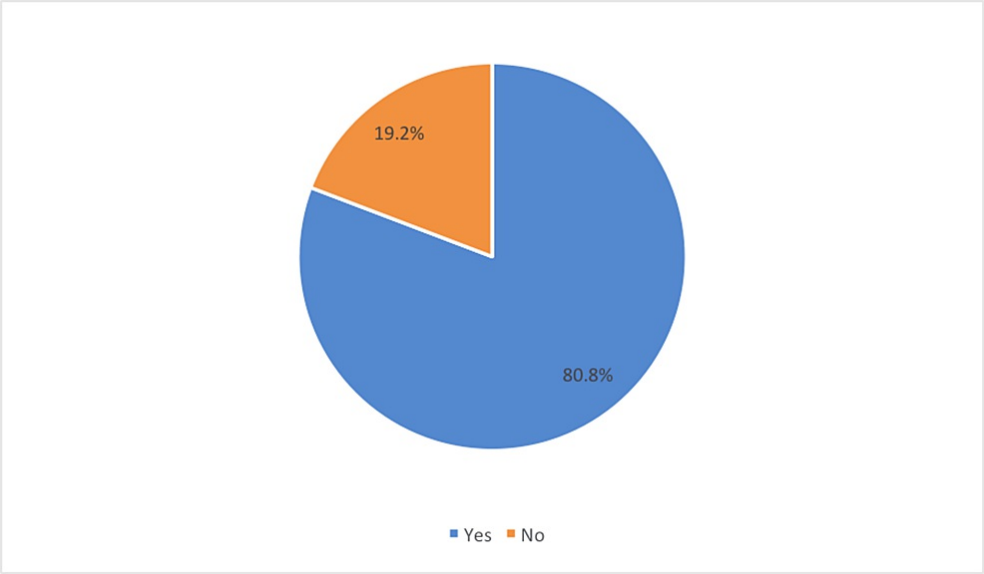
. Usage of artificial intelligence (AI)-enabled virtual assistants (VAs) by the participants

Among the total participants, 80.8% had used AI-enabled VAs for treatment and decision-support as shown in Figure 1.

**Figure 2:**
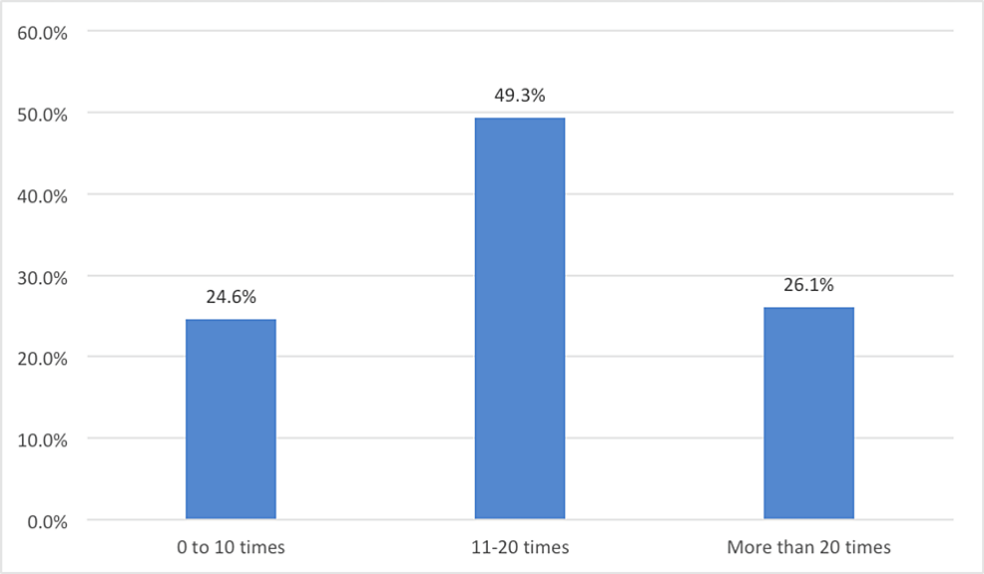
Frequency of artificial intelligence (AI)-enabled virtual assistants (VAs) by participants in the previous month (N=321)

Out of the 80.8% (321 participants) who used AI-enabled VAs, 49.3% used 11-20 times and 26.1% used more than 20 times in the last month indicating normal to high usage rates (Figure 2).

The results (Table 2) pertaining to the acceptance of AI-enabled VAs by leukemia patients for managing their condition provide a comprehensive view of the various factors influencing their willingness to integrate this technology into their healthcare journey. The mean scores highlight several important insights. Firstly, the relatively high mean score for PE at 3.14 suggests that leukemia patients perceive AI-enabled VAs as promising tools that can significantly benefit their disease management, indicating a favorable attitude towards their utility. EE also scored above 3, with a mean of 3.05, implying that patients find the use of AI-enabled VAs to be relatively convenient and manageable. This suggests that the ease of use and integration into their healthcare routines is not a significant hindrance.

**Table 2:** Mean ratings of various factors influencing acceptance of artificial intelligence (AI)-enabled virtual assistants (VAs)

Factors	Mean (μ)
Performance expectancy (PE)	3.14
Effort expectancy (EE)	3.05
Social influence (SI)	2.76
Facilitating conditions (FC)	3.11
Behavioral intention (BI)	2.97
Trust	2.91
Perceived privacy risk (PPR)	2.44
Personalized innovativeness (PI)	3.14

Conversely, SI scores were lower at 2.76, indicating that external factors such as the influence of friends, family, or healthcare providers might not be substantial drivers in the acceptance of AI-enabled VAs among leukemia patients. FC with a mean score of 3.11 implies that patients feel adequately supported and have access to the resources required for effectively utilizing AI-enabled VAs, which is a positive sign for their adoption. BI at 2.97 and trust at 2.91 reveal that while patients may have a generally positive disposition toward AI-enabled VAs, they may still harbor reservations and uncertainties that could impact their actual intent to use these technologies. The PPR score of 2.44 indicates significant concerns regarding the protection of personal medical data. Addressing these concerns is crucial for building patient trust and fostering acceptance. Finally, PI with a mean of 3.14 suggests that patients are open to innovative, personalized solutions in their leukemia management, which aligns with the potential of AI technology to provide tailored care.

Table 3 presents a detailed analysis of gender differences in the factors influencing the acceptance of AI-enabled VAs for leukemia management. Notably, males exhibit notably higher levels of perceived PE, with an average score of 3.44, compared to females who scored an average of 2.71. This stark difference is supported by a significant t-value of 7.30, with a p-value less than 0.0001, indicating that males are more confident in the utility of AI-enabled VAs for managing their condition. Similarly, males also report higher EE with an average score of 3.34, compared to females who scored 2.65, with a high t-value of 7.25 and a p-value less than 0.0001. These findings suggest that males find using AI-enabled VAs for leukemia management to be more convenient and less burdensome than females. In contrast, gender differences are less pronounced in other factors such as SI and PPR, where the p-values are not statistically significant, indicating that males and females have relatively similar perceptions regarding these aspects. However, males scored significantly higher in FC, BI, trust, and PI, underlining a greater readiness among males to adopt AI-enabled VAs for leukemia management.

**Table 3:** Differences in participants’ perceptions related to factors influencing acceptance of artificial intelligence (AI)-enabled virtual assistants (VAs) by gender PE: Performance expectancy, EE: Effort expectancy, SI: Social influence, FC: Facilitating conditions, BI: Behavioral intention, PPR: Perceived privacy risk, PI: Personalized innovativeness, * Statistically significant difference; df: degrees of freedom; SD: Standard Deviation

Factors	Gender	N	Mean	SD	df	T-value	p-value
PE	Male	233	3.44	.69	292	7.3024	< .0001*
	Female	164	2.71	1.16			
EE	Male	233	3.34	.56	274	7.2508	< .0001*
	Female	164	2.65	1.12			
SI	Male	233	2.81	1.17	354	1.3274	.0926
	Female	164	2.67	1.14			
FC	Male	233	3.45	.73	303	8.0574	< .0001*
	Female	164	2.63	1.12			
BI	Male	233	3.3	.96	319	7.4144	< .0001*
	Female	164	2.49	1.27			
Trust	Male	233	3.22	.85	329	7.7263	< .0001*
	Female	164	2.45	1.04			
PPR	Male	233	2.21	.73	298	5.6561	< .0001*
	Female	164	2.78	1.17			
PI	Male	233	3.23	.89	319	4.1848	< .0001*
	Female	164	2.88	1.18			

The data in Table 4 highlights distinct differences between age groups in their perceptions of various factors related to the acceptance of AI-enabled VAs for leukemia management. Firstly, in terms of PE, individuals aged 40 and younger (<=40 years) exhibit a significantly higher mean score of 3.23, indicating a more positive outlook on the utility of AI-enabled VAs, compared to individuals over 40 years, who have a lower mean score of 2.88. This discrepancy is supported by a t-value of 2.98 and a p-value of 0.0016, underlining a statistically significant difference in the perceived benefits of AI-enabled VAs. It suggests that younger individuals are more optimistic about the advantages of AI technology for leukemia management.

**Table 4:** Differences of participants’ perceptions related to factors influencing acceptance of artificial intelligence (AI)-enabled virtual assistants (VAs) by age PE: Performance expectancy, EE: Effort expectancy, SI: Social influence, FC: Facilitating conditions, BI: Behavioral intention, PPR: Perceived privacy risk, PI: Personalized innovativeness, * Statistically significant difference; df: degrees of freedom; SD: Standard Deviation

Factors	Age	N	Mean	SD	df	T-value	p-value
PE	<=40 years	298	3.23	.97	165	2.9848	.0016*
	> 40 years	99	2.88	1.05			
EE	<=40 years	298	3.14	.9	175	3.0408	.0013*
	> 40 years	99	2.81	.85			
SI	<=40 years	298	2.74	1.24	195	.59672	.2756
	> 40 years	99	2.81	.92			
FC	<=40 years	298	3.19	.99	162	2.89114	.0021*
	> 40 years	99	2.85	1.11			
BI	<=40 years	298	3.04	1.28	123	2.28954	.0115*
	> 40 years	99	2.75	1.09			
Trust	<=40 years	298	3.09	.98	172	6.61074	< .0001*
	> 40 years	99	2.34	.95			
PPR	<=40 years	298	2.36	.94	161	2.63574	.0046*
	> 40 years	99	2.67	1.07			
PI	<=40 years	298	3.27	.91	148	3.94822	< .0001*
	> 40 years	99	2.77	1.29			

Secondly, in EE, the <=40 years group demonstrated a higher mean score of 3.14, while the > 40 years group had a lower mean score of 2.81. This distinction is substantiated by a t-value of 3.04 and a p-value of 0.0013, indicating a significant divergence in the convenience and ease of using AI-enabled VAs for leukemia management. Younger individuals find these tools more user-friendly and less burdensome compared to their older counterparts. In other factors, such as SI and PPR, the differences between the two age groups were not statistically significant, with p-values greater than 0.05. This suggests that both age groups have relatively similar perceptions regarding the influence of social factors and privacy concerns. However, age-related disparities are significant in FC, BI, trust, and PI. Younger individuals exhibit higher scores in these factors, signifying greater readiness to adopt AI-enabled VAs for leukemia management.

The correlation matrix (Table 5) reveals several important relationships between the factors influencing the acceptance of AI-enabled VAs for leukemia management. Notably, PE demonstrates meaningful correlations with several other factors. It exhibits a moderate positive correlation with EE at 0.47, suggesting that as patients perceive AI-enabled VAs to be more beneficial, they also tend to find them easier to use. Furthermore, PE shows a similar positive correlation with FC at 0.49, indicating that the perception of AI's benefits is associated with the availability of necessary resources for its use. Most significantly, PE has a strong positive correlation of 0.62 with BI, emphasizing that as performance expectations increase, the intention to adopt AI-enabled VAs for leukemia management also rises. These findings underscore the importance of enhancing the perceived performance benefits of AI-enabled VAs to positively influence user intentions and the overall acceptance of this technology in healthcare.

**Table 5:** Correlation matrix PE: Performance expectancy, EE: Effort expectancy, SI: Social influence, FC: Facilitating conditions, BI: Behavioral intention, PPR: Perceived privacy risk, PI: Personalized innovativeness

	PE	EE	SI	FC	Trust	PPR	PI	BI
PE	1							
EE	0.47039	1						
SI	0.31437	0.43415	1					
FC	0.48868	0.3278	0.12208	1				
Trust	0.16922	0.21699	0.01124	0.20105	1			
PPR	-0.1207	-0.0793	-0.0647	-0.1696	-0.0607	1		
PI	0.14251	0.10237	-0.0722	0.20341	0.22539	-0.1466	1	
BI	0.61875	0.46563	0.27607	0.46803	0.1584	-0.1268	0.04993	1

Additionally, the matrix shows several other noteworthy correlations, such as the positive correlation between trust and PE (0.17) and FC (0.20), indicating that trust in AI is related to performance expectations and the availability of resources. Conversely, there is a negative correlation between PPR and several factors, suggesting that higher privacy concerns are associated with lower acceptance of AI. These correlations provide valuable insights for healthcare providers and policymakers aiming to promote AI adoption while addressing user concerns.

## Discussion

The purpose of this study is to analyze the various influencing factors affecting the adoption of AI-enabled VAs for self-management of leukemia. Accordingly, different factors with varying levels of impact were identified in this study. Firstly, more than two-thirds of the participants have been using various AI-enabled VAs such as ChatGPT and Google Bard for managing their condition. Furthermore, two-thirds of them used AI-enabled VAs more than 11 times in the last month. This progress indicates the gradual rise in the use of AI-enabled VAs in the self-management of leukemia, reflecting similar results for various chronic conditions in recent studies [22-26].

In assessing the various factors, it can be observed that PE and PI were found to be major positive influencing factors implying the patients' beliefs about AI-enabled VAs as effective and promising tools and are open to innovate the various solutions for managing their condition. In addition, both SI and trust were identified to be moderately influencing the participants on the adoption of AI-enabled VAs. Similar findings can be observed in studies [27-30], which highlighted that the perceived PE, FC, EE, and PI were identified to be strongly associated with the adoption of AI-enabled VAs. However, PRR achieved a low mean rating, indicating the participants' concerns over their personal data in similar to the studies [31,32].

Focusing on the gender differences, it is observed that males perceived AI-enabled VAs to be effective, easy to use, trustable, and enhance innovativeness compared to females. These differences may be due to the novel nature of AI and unawareness of its potential application among certain population groups [33-36]. However, no significant differences between SI and PPR were observed, indicating the common concerns of privacy across both genders. These gender disparities in the acceptance of AI assistants highlight the importance of tailoring interventions and educational strategies to address the specific needs and concerns of both male and female leukemia patients.

In relation to differences in perceptions among the participant age groups, significant differences were observed between the older and younger participants in relation to all factors except SI. While younger participants perceived higher PE, EE, FC, BI, trust, and PI, older participants perceived higher SI and PPR compared to their counterparts as observed in various studies [37-39]. This underscores the importance of tailoring strategies to address the unique needs and concerns of both age groups when implementing AI technologies in healthcare.

Upon doing an analysis of the correlations among the components, it becomes evident that a robust positive association has been established between PE and BI, signifying that an increase in PE corresponds to a higher inclination to utilize AI-enabled VAs [40]. Moreover, there exists a moderate positive correlation between PE and EE, suggesting that an increase in performance expectations is associated with a corresponding increase in the impression of ease of use. This finding indicates that individuals who hold higher expectations regarding the efficacy of AI technology also exhibit a greater ease of utilization. Conversely, PPR exhibits a negative correlation with certain parameters. The PPR variable exhibits negative associations with the PE, EE, SI, FC, and trust variables. This suggests that when individuals perceive a higher level of privacy risk, various elements connected to acceptance, including performance expectations, ease of use, social influence, environmental support, and trust, tend to exhibit a reduction. Patients who exhibit a higher level of anxiety regarding privacy may consequently demonstrate a reduced level of acceptance towards AI-enabled assistants. In conclusion, the aforementioned connections elucidate the intricate dynamics among many components and emphasize the significance of addressing privacy apprehensions in order to foster acceptance among familial patients.

Implications

This study on the influencing factors of AI-enabled virtual assistant adoption for leukemia management offers valuable theoretical and practical implications. It contributes to the growing healthcare literature by highlighting the multifaceted nature of AI acceptance, emphasizing the variable impact of factors like PE and PI across different populations. The study's findings have practical significance for healthcare stakeholders. Healthcare providers and policymakers can use these insights to shape the implementation of AI-enabled VAs, focusing on tailored strategies to address patient concerns. Privacy and security measures must be robust to build trust and mitigate privacy risks, while gender and age disparities necessitate interventions that cater to unique needs. Promoting AI literacy among patients is crucial, given the impact of knowledge awareness, enabling them to make informed decisions about AI adoption and ultimately improving leukemia management and healthcare delivery.

Limitations

Despite the valuable insights provided by this study, several limitations need to be acknowledged. Firstly, the research is based on self-reported data from leukemia patients, which may be subject to response bias or social desirability bias, potentially affecting the accuracy of the results. Secondly, the study primarily focuses on patients from public hospitals and self-help communities, which may not represent the broader population of leukemia patients, particularly those with different socioeconomic backgrounds and healthcare access. Additionally, the study does not explore the specific characteristics of AI-enabled virtual assistants used, which can vary widely in terms of capabilities and features, potentially influencing patient perceptions. Furthermore, as the study is cross-sectional, it provides a snapshot of patient attitudes at a specific point in time, and longitudinal research could offer a more comprehensive understanding of the evolving acceptance of AI in leukemia management. Lastly, while the study identifies gender and age disparities, it does not delve into the underlying reasons for these differences, which could be a valuable area for further investigation.

## Conclusions

In conclusion, this study offers valuable insights into the influencing factors of AI-enabled virtual assistant adoption for self-help and leukemia management. The research highlights the multifaceted nature of patient acceptance, shedding light on the variable impact of factors such as PE, EE, SI, FC, trust, PPR, and PI. These findings contribute to the evolving body of literature on AI adoption in healthcare. From a practical perspective, healthcare stakeholders can leverage these insights to inform the implementation of AI-enabled virtual assistants. Tailored strategies and interventions should be designed to address the unique concerns and preferences of different patient segments, considering gender and age disparities. Additionally, the importance of robust privacy and security measures cannot be overstated, as these are crucial for building trust and mitigating privacy concerns. Promoting AI literacy among patients is essential, empowering them to make informed decisions about AI adoption and ultimately improving leukemia management and healthcare delivery. While this study has its limitations, it lays the foundation for further research in this critical area, with the potential to transform leukemia management and patient care through AI-enabled solutions.
